# Translating and validating the hoarding rating scale-self report into Arabic

**DOI:** 10.1186/s40359-023-01277-1

**Published:** 2023-08-16

**Authors:** Nour Mohammad Hussain, Dalal Hasan AlMansouri, Muneera AlGhareeb, Yaser Mansoor Almutawa, Omaima Khaled Bucheeri, Mai Helmy, Khaled Trabelsi, Zahra Saif, Haitham Jahrami

**Affiliations:** 1 College of Medicine and Medical Sciences, Arabian Gulf University, Manama, Bahrain; 2 Psychology Department, College of Education, Sultan Qaboos University, Muscat, Oman; 3 Psychology Department, Faculty of Arts, Menoufia University, Shibin Al Kawm, Egypt; 4 High Institute of Sport and Physical Education of Sfax, University of Sfax, Sfax, 3000 Tunisia; 5 Research Laboratory Education, Motricity, Sport and Health, EM2S, LR19JS01, University of Sfax, Sfax, 3000 Tunisia; 6 Government Hospitals, Manama, Bahrain

**Keywords:** Hoarding disorder, Assessment, Scale, Arabic, Psychometrics

## Abstract

**Background:**

Hoarding disorder is characterized by a persistent inability to part with possessions due to a perceived need to keep them, regardless of their actual value. Arabic-speaking populations currently lack a validated tool specifically designed to assess hoarding symptoms.

**Objective:**

This study aimed to translate, adapt, and validate the Hoarding Rating Scale-Self Report (HRS-SR) into the Arabic-language.

**Methods:**

The study employed the gold standard approach to translation, involving forward translation by independent translators and back translation review. We conducted a cross-sectional study using an online survey completed by 500 participants from four Arabic-speaking countries. Psychometric analyses included internal consistency, test-retest reliability, convergent validity against generalized anxiety disorder (GAD-7), and confirmatory factor analysis.

**Results:**

With a McDonald’s omega and Cronbach’s alpha of approximately 0.80, the Arabic translation of the HRS-SR showed acceptable test-retest reliability as well as good internal consistency. The survey also showed strong convergent validity with the 7-item survey for GAD-7. Confirmatory factor analysis supported a one-factor structure, confirming that each item measured the same construct.

**Conclusion:**

The HRS-SR is a trustworthy and valid tool for evaluating hoarding symptoms in Arabic-speaking people. This survey could be helpful for both clinical and academic research. Future research should examine cultural variations in hoarding behavior in Arabic-speaking populations and validate the questionnaire in clinical populations.

## Introduction

Regardless of their actual value, possessions can be difficult to part with or discard in cases of hoarding disorder, a mental health condition [1]. Clutter can cause serious distress, impairment, and dysfunction when it accumulates as a result of this behavior [2]. The Diagnostic and Statistical Manual of Mental Disorders, Fifth Edition (DSM-5) recognizes hoarding disorder as a separate diagnosis [3].

Hoarding behavior has been observed across various cultures and countries [4]. A recent systematic review and meta-analysis revealed that hoarding disorder had a pooled estimated prevalence of 2.5% (confidence interval (CI) : 1.7–3.6%), and subgroup analysis showed that prevalence rates were comparable for both males and females [4].

Hoarding behavior appears to be widespread in Arab cultures, according to anecdotal data [5]. However, due to the lack of validated Arabic-language tests, research on hoarding disorder in Arab nations is scarce.

The value or significance of hoarding practices in Arab culture may stem from a number of factors. For instance, some people could give a lot of importance to their items as a status or wealth signal [6, 7]. This might be especially true in cultures where material items are used as measures of success and accomplishment [6, 8]. Hoarding tendencies can occasionally be linked to worry or future-related fear [9]. Some people may feel the urge to hold onto material possessions in a region that has witnessed socio-political, economic and international emergencies (e.g., COVID-19) in order to feel secure or ready for any future challenges [10].

Social isolation, health issues, an increased risk of fire, and other dangers are just a few of the detrimental effects of the disorder [11]. Treatment for hoarding is a complex and difficult condition, and research is ongoing in this area [1]. Our understanding of hoarding has advanced, along with improvements in hoarding diagnosis and treatment. This progress has been driven by the development of reliable assessment tools such as the Hoarding Rating Scale-Self Report (HRS-SR) [12].

The HRS-SR was developed as a self-report tool to evaluate hoarding disorder symptoms [12]. Five items make up the HRS-SR, which offers a thorough evaluation of hoarding-related behaviors and distress [12]. The questionnaire includes questions that measure emotional distress associated with these behaviors as well as clutter, difficulty discarding, difficulty acquiring, and excessive saving of items [12]. On a scale from 0 (not at all) to 8 (extreme), respondents rate the frequency and severity of each behavior [12]. A total score is calculated by summing the five HRS-SR questions, resulting in a range from 0 to 40, with higher values denoting more severe hoarding symptomatology [12]. The HRS-SR has demonstrated good internal consistency, test-retest reliability, and convergent validity with other hoarding symptomatology measures [12]. Researchers and clinicians can use the HRS-SR to evaluate hoarding symptoms in individuals because it is simple to administer and score [12]. Overall, the HRS-SR makes a significant contribution to the evaluation of hoarding disorder and can be used to guide treatment planning and long-term progress monitoring [12].

This purpose of this paper is to describe the translation and validation of the HRS-SR into Arabic language, aiming to support clinicians and researchers in Arabic-speaking communities with the assessment of hoarding symptoms in individuals.

## Methods

### The translation process

The translation and back translation of the HRS-SR adhered to industry best practices in order to guarantee its accuracy and cultural appropriateness. The translation process involved five steps [13]. First, the translation from English into Arabic: The questionnaire was initially translated from English into Arabic by two independent translators who were both fluent in both languages. They were told to create a translation that is both culturally proper for Arabic-speaking people and accurately reflects the meaning of each item in the questionnaire. Second, the synthesis: After comparing their translations, the two translators reached a consensus on the questionnaire’s final Arabic version. They conducted a thorough review of each item to ensure linguistic clarity, accuracy, and cultural and religious appropriateness. Third, the final Arabic translation of the questionnaire was back translated into English by two independent translators who were not involved in the first stage and were fluent in both English and Arabic. The questionnaire’s original English version remained hidden from the back-translators. Fourth, a bilingual expert then compared the two back-translations to the questionnaire’s original English version to spot any discrepancies or mistakes. To ensure that the Arabic version accurately reflected the meaning of the original English version, any differences were discussed with the translators. To ensure the accuracy and consistency of the translation process, we computed Cohen’s kappa coefficient [14] and intraclass correlation coefficient (ICC) [15], which are frequently used statistical techniques for evaluating the level of agreement between two or more raters. For each question in the questionnaire, we specifically calculated the kappa coefficient or ICC to find the degree of agreement between the two translators. Results showed that both Cohen’s kappa coefficient and ICC were > 0.99, suggesting a perfect match between translations. Fifth, the pilot testing: To make sure that the wording was clear and understandable, the Arabic version of the questionnaire was then pilot tested on a small sample of Arabic-speaking people. The pilot study sample involved 25 participants who were acquaintances or family members of the research team. The questionnaire remained unchanged during the pilot study, and the same version was used for the main study.

### Data collection

The study was promoted on a variety of social media platforms, including Instagram, Facebook, Discord, and Twitter, as well as instant messaging services such as WhatsApp, Viber, and Signal, through our research network across Bahrain, Egypt, Jordan and Tunisia. Participants were directed to an online survey of the HRS-SR in the Arabic language, which was presented through a Google form created by the first author. Prior to commencing the survey, participants had to supply informed consent and respond to basic demographic questions, such as age, gender, and country of residence. To assess test-retest reliability, a subsample of participants (n = 100) received the questionnaire twice, with a two-week interval between administrations.

Anxiety disorders and hoarding have a close relationship [16]. In fact, hoarding disorder was conventionally regarded as a manifestation of obsessive-compulsive disorder (OCD), which is a type of anxiety disorders [17, 18]. Making judgments about what to retain and what to throw away can be stressful for people with hoarding disorder, and sometimes they find comfort or relief in collecting or saving things [17]. Thus, in the present study, for validation purposes, the correlation between hoarding symptoms and anxiety symptoms was examined using the Generalized Anxiety Disorder 7-item (GAD-7) questionnaire [19]. It has been demonstrated that the GAD-7 has good psychometric properties in a variety of populations, making it a widely used measure of anxiety symptoms [19]. Additionally, the Arabic language adaptation of the GAD-7 has adequate psychometric properties with Cronbach alpha of about 0.80 [20]. The Japanese version of GAD-7 was successfully used previously to validate the Japanese version of the HRS-SR [21].

For the validation of the Arabic version of the HRS-SR questionnaire, a sample size of 385 participants would be adequate for factor analysis, which is typically 5–10 participants per questionnaire item [22], and would offer sufficient statistical power of about 80% for the factor analysis and other psychometric analyses used in this study. To increase the power to 90% for the final analyses, the study aimed for 500 participants.

### Ethics

The study procedures adhered to the ethical guidelines outlined in the Helsinki Declaration of 1964 and its later amendments (1975, 1983, 1989, and 1996). The research received approval from the Institutional Review Board at the University of Menoufia in Menoufia, Egypt. Participation in the study was entirely voluntary, and participants had the right to withdraw from the study at any time without penalty.

### Statistical analyses

In this study, descriptive statistics were used to describe the characteristics of the sample and summarize the results. The mean was used as a measure of central tendency, and the standard deviation was used as a measure of variability. Frequencies and percentages were also used to describe the distribution of responses for categorical variables. We compared the scores on the HRS-SR between males and females, as well as between two distinct age groups: individuals under the age of 35 and those aged 35 years or older. To assess for differences in means between these groups, we used independent samples t-tests.

The suite for statistical computation and visualization offered by the R Statistical Foundation (R version 4.2.2) was used for all analyses. The threshold for statistical significance in all analyses was set at a p-value of 0.05 or lower.

The internal consistency of the HRS-SR in Arabic was assessed using two measures: McDonald’s omega [23] and Cronbach’s alpha [23]. The internal consistency value > 0.75 [23], which is regarded as a reliable level of consistency, was set as the threshold for acceptable internal consistency. We also looked into whether any items that had weak correlations with the other items during the analysis could be removed from the questionnaire. However, no items were eliminated from this study because all the items showed high internal consistency, suggesting that they were all measuring the same construct and enhancing the validity of the questionnaire.

Using Pearson’s correlation coefficient [24], which gauges the strength and direction of a relationship between two variables, the correlation between the HRS-SR and the GAD-7 was investigated to examine convergent validity.

The Arabic version of the HRS-SR questionnaire’s factor structure was investigated using confirmatory factor analysis (CFA) [25]. CFA is a statistical technique that enables researchers to examine the degree to which the observed data conform to a proposed theoretical framework [25]. In this study, a one-factor model—which postulates that all of the questionnaire’s items are measuring the same construct—was the hypothesized structure [25].

The data were examined using a structural equation modeling (SEM) strategy in order to conduct the CFA [26]. The maximum likelihood estimation method, an often-employed technique for SEM, was used to conduct the CFA. The comparative fit index (CFI), Tucker-Lewis index (TLI), root mean square error of approximation (RMSEA), and standardized root mean square residual were the fit indices used to assess the model’s goodness of fit (SRMR) [27].

## Results

This research involved 500 participants. The majority of participants (85%) belonged to the age category below 35 years, and 64% were female. The mean HRS-SR score was 13.00 ± 7.50 for the entire sample. Females had a higher HRS-SR score compared to males (13.39 ± 7.44 vs. 12.44 ± 7.59); however, this difference did not reach statistical significance (p = 0.18). Similarly, no statistically significant difference was observed based on age at the cutoff of 35 years (i.e., < 35 years of age = 13.04 ± 7.50 vs. ≥ 35 years of age = 12.91 ± 7.59, p = 0.88).

To determine the accuracy of the scale, McDonald’s omega was calculated. The result was 0.785 (95%CI 0.755–0.815), which denotes an acceptable level of internal consistency. Cronbach’s alpha was also calculated, and the resultant value of 0.781 (95%CI 0.748–0.809), further affirming the scale’s acceptable level of internal consistency. No item was suggested for deletion to improve McDonald’s omega or Cronbach’s alpha. Detailed results are shown in Table 1.

Correlations with the Generalized GAD-7 questionnaire was examined to ascertain the validity of the Arabic version of the questionnaire. The HRS-SR Arabic version has good convergent validity, as shown by the significant correlation between the two questionnaires (r = 0.67, p < 0.01).

Participants who completed the questionnaire twice demonstrated a significant intraclass correlation coefficient of r = 0.88 (p < 0.01), indicating acceptable test-retest reliability.

Confirmatory factor analysis (CFA) was conducted to investigate the factor structure of the Arabic translation of the HRS-SR questionnaire. Maximum likelihood estimation was used for the CFA, and fit indices including the CFI, TLI, RMSEA, and SRMR were examined. The one-factor model had a respectable fit, according to the CFA results, which included a CFI of 0.96, TLI of 0.92, RMSEA of 0.09, and SRMR of 0.04. Detailed results shown in Table 2.

## Discussion

In this study, we aimed to evaluate the psychometric properties of the HRS-SR through a rigorous validation process. Our findings provide strong evidence supporting the reliability and validity of the HRS-SR questionnaire, establishing it as a valuable tool for researchers investigating hoarding disorder across diverse domains. Our analyses demonstrated that the HRS-SR exhibits high internal consistency, with Cronbach’s alpha values exceeding the recommended threshold of 0.70.

In line with the evidence for reliability (internal consistency), we also found support for the HRS-SR construct validity. CFA confirmed that the hypothesized factor structure fit the data well, with fit indices (CFI, TLI, RMSEA, and SRMR) meeting or surpassing their respective benchmarks. This suggests that the HRS-SR effectively measures an important and well-defined construct.

The psychometric validation of the HRS-SR has several important implications for researchers and practitioners. The research findings suggest that the Arabic version of the HRS-SR is highly reliable and valid. Overall, the psychometric analyses of the Arabic version of the HRS-SR showed that it had a one-factor structure, good convergent validity, high internal consistency, and acceptable test-retest reliability. These results suggest that the questionnaire’s Arabic translation is a valid and trustworthy tool for evaluating hoarding symptoms in Arabic-speaking people.

The mean HRS-SR score in the present study appears to be higher than the score of the general population in Switzerland 2.83 (SD = 4.20) [28], USA 3.86 (SD = 5.90) [29], and Japan 9.99 (SD = 8.98) [21]. However, our results appeared to be lower compared to teenagers in Sweden 19.7 (SD = 4.9) [30].

For a number of reasons, hoarding disorders warrant further study [1, 2, 31]. First off, hoarding disorder is a relatively common psychiatric condition that can cause sufferers to feel extremely distressed and impaired [32]. The lives of those who suffer from hoarding disorder can be improved by understanding its causes, risk factors, and effective treatments [33]. Second, hoarding behavior can seriously endanger both the environment and the people who engage in it [34]. In addition to increasing the risk of fire, falls, and other dangers, hoarding can help infectious diseases spread [1, 2, 31, 35]. Identifying effective hoarding interventions can therefore help to reduce these risks and advance public health and safety [36].

Hoarding behavior frequently co-occurs with other psychiatric disorders like obsessive-compulsive disorder, depression, and anxiety [1, 2, 31, 35]. Improved diagnostic precision and treatment outcomes for these patients can result from an understanding of the connection between hoarding and these other conditions [37]. Overall, research into hoarding disorder is essential to deepening our knowledge of this complex disorder and creating potent treatments for those who suffer from it [38].

### Strengths and limitations

The HRS-SR questionnaire’s Arabic translation and validation have a number of strengths. First, a rigorous translation procedure was implemented, following best practices to ensure the questionnaire’s accuracy and cultural appropriateness. This approach guarantees that the Arabic version accurately reflects the meaning of the original English version of the questionnaire. Second, comprehensive psychometric evaluations were conducted, including assessments of internal consistency, convergent validity, test-retest reliability, and CFA. These thorough evaluations offered solid proof of the validity and dependability of the Arabic version of the survey.

The HRS-SR questionnaire’s Arabic translation and validation also have certain limitations. First, convenience sampling was employed using social media and instant messaging platforms. This sampling method may have introduced selection bias and, consequently, limited the generalizability of the results. Second, self-report measures in the study may be susceptible to social desirability bias and response bias. Third, in the Japanese validation of the HRS-SR Tsuchiyagaito and colleagues [21] stated that the correlations with GAD-7 was weaker than that with Japanese Savings Inventory-Revised (SI-R), suggesting that the HRS measures a more similar construct as the SI-R than it does the GAD-7. This may suggest that the GAD-7 was actually considered an appropriate measure of divergent validity than convergent. It is recognized that there is currently no Arabic measure of hoarding symptoms, making convergent validity with a direct measure difficult. Fourth, no clinical diagnosis of hoarding disorder was included in the study, which may have limited the generalizability of the results to clinical populations. Fifth, the study’s modest sample size limited the ability to detect potential questionnaire issues.

## Conclusion

In conclusion, the HRS-SR in Arabic is a valid and reliable tool for evaluating hoarding symptoms in Arabic-speaking people. Our results show potentially very good internal consistency, very good test re-test, and good convergent validity, though this conclusion is limited by lack of other validated measures of hoarding symptoms in the Arabic language. Further research is also needed to examine cultural variations in hoarding behavior and attitudes in Arabic-speaking populations. In the Arabic-speaking world, better diagnosis and treatment of hoarding disorder may result from an understanding of these cultural factors. Fundamentally, the HRS-SR appear to be a workable tool for evaluating hoarding symptoms in Arabic-speaking people and may be helpful for both research and clinical purposes.

**Table 1:** Hoarding Rating Scale Reliability Statistics

Estimate	McDonald’s ω	Cronbach’s α
Point estimate	0.785	0.781
95% CI lower bound	0.755	0.748
95% CI upper bound	0.815	0.809
**If item dropped**		
HRS-SR - Item #1	0.741	0.733
h-SR - Item #2	0.742	0.733
h-SR - Item #3	0.760	0.754
h-SR - Item #4	0.714	0.709
h-SR - Item #5	0.769	0.767

**Table 2:** Confirmatory Factor Analysis of the Hoarding Rating Scale-Self Assessment

Fit indices	Value
Comparative Fit Index (CFI)	0.964
Tucker-Lewis Index (TLI)	0.927
Bentler-Bonett Non-normed Fit Index (NNFI)	0.927
Bentler-Bonett Normed Fit Index (NFI)	0.956
Parsimony Normed Fit Index (PNFI)	0.478
Bollen’s Relative Fit Index (RFI)	0.913
Bollen’s Incremental Fit Index (IFI)	0.964
Relative Noncentrality Index (RNI)	0.964
Root mean square error of approximation (RMSEA)	0.095
RMSEA 90% CI lower bound	0.063
RMSEA 90% CI upper bound	0.131
RMSEA p-value	0.013
Standardized root mean square residual (SRMR)	0.036
Hoelter’s critical N (α = 0.05)	200.267
Hoelter’s critical N (α = 0.01)	272.551
Goodness of fit index (GFI)	0.978
McDonald fit index (MFI)	0.977
Expected cross validation index (ECVI)	0.096
Log-likelihood	-5055.32
Number of free parameters	10
Akaike (AIC)	10130.63
Bayesian (BIC)	10172.78
Sample-size adjusted Bayesian (SSABIC)	10141.04
Log-likelihood	-5055.32
*Χ*²	27.778; p < 0.001

## Data Availability

The data that support the findings of this study are available from the corresponding author based upon request.
